# Stimulation of the Tibial nerve Repetitively to Improve Incontinence in Parkinson’s Electronically (STRIPE trial): a randomised control trial of tibial nerve stimulation for bladder symptoms in Parkinson’s disease using a self-contained wearable device

**DOI:** 10.1186/s13063-022-06827-3

**Published:** 2022-10-28

**Authors:** Matthew D. Smith, Emma Tenison, Marcus J. Drake, Yoav Ben-Shlomo, Emily J. Henderson

**Affiliations:** 1 Older People’s Unit, Royal United Hospital NHS Foundation Trust, Bath, UK; 2 Population Health Sciences, Bristol Medical School, University of Bristol, Bristol, UK; 3 Bristol Urological Institute, North Bristol NHS Trust, Bristol, UK; 4 Department of Surgery and Cancer, Imperial College, London, UK

**Keywords:** Bladder symptoms, Parkinson’s disease, Neuromodulation, Tibial nerve stimulation, Randomised control trial

## Abstract

**Background:**

Bladder symptoms are common in Parkinson’s disease (PD), affecting half of all individuals. These have significant impact on quality of life as well as implications for morbidity, contributing to falls and hospital admission. The treatment of bladder symptoms can be complicated by the tendency to side-effects in people with PD including cognitive impairment and gait instability with anti-muscarinics. The development of new, better treatments is therefore warranted. Tibial nerve stimulation is a form of neuromodulation demonstrated to improve overactive bladder symptoms in non-neurogenic cohorts. Previously requiring hospital attendance, we aim to explore the use of this intervention using a simple device that can be used by patients at home.

**Methods:**

STRIPE is a phase II randomised control trial of tibial nerve stimulation delivered by the Geko™ device, a small, self-adhesive neuromuscular stimulation device currently used for thromboembolism prophylaxis post-surgery. Active tibial nerve stimulation will be compared to sham stimulation, with participants blinded to treatment allocation and undertaking outcome assessment whilst still blinded. Participants will be asked to self-administer stimulation at home twice per week, for 30 min per session, over the course of 3 months. Primary outcome measure will be the International Consultation on Incontinence Overactive Bladder Questionnaire (OAB) at week 12. Secondary outcomes will include pre- and post-intervention bladder diary (frequency, urgency episodes, nocturia), patient perception of global change, bowel function and bladder-related quality of life. Participants will be recruited from the Proactive Integrated Management and Empowerment (PRIME) cross-sectional trial in which participants have been screened for bladder symptoms and invited to take part, as well as clinician referral from around the region.

**Discussion:**

This trial will involve a randomised control trial of a novel and easy to use method of delivering tibial nerve stimulation for PD in the patient’s own home. This may potentially have huge benefit, avoiding the problems with side effects that can be seen with anti-muscarinics and providing a new potential modality of treatment.

**Trial registration:**

ISRCTN11484954. Registered on 22 June 2021.

## Administrative information

**Table:** 

**Title {1}**	Stimulation of the Tibial nerve Repetitively to Improve Incontinence in Parkinson’s Electronically (STRIPE trial): a randomised control trial of tibial nerve stimulation for bladder symptoms in Parkinson’s disease using a self-contained wearable device
Trial registration {2a and 2b}	ISRCTN11484954. Prospectively registered 22/06/21
Protocol version {3}	Version 3.0 08/02/22
Funding {4}	The Gatsby Charitable Foundation
Author details {5a}	1)Older people’s unit, Royal United Hospital NHS Foundation Trust, Bath, UK 2)Population Health Sciences, Bristol Medical School, University of Bristol, Bristol, UK 3)Bristol Urological Institute, North Bristol NHS Trust, Bristol, UK 4) Translational Health Sciences, Bristol Medical School, University of Bristol, Bristol, UK
Name and contact information for the trial sponsor {5b}	University of Bristol: Senate House, Tyndall Avenue, Bristol BS8 1TH research-governance@bristol.ac.uk
Role of sponsor {5c}	Neither sponsor or funder have been involved with trial design. The University of Bristol undertakes research governance to ensure satisfactory conduct of research

## Introduction

### Background and rationale {6a}

Parkinson’s disease is the second most common neurodegenerative disorder, affecting one in 37 people during their lifetime [1]. A recent large-scale study suggests up to 57% of individuals with Parkinson’s (PD) report bladder symptoms [2]. These have a profoundly negative impact on quality of life [3] and have been identified as a priority area for research by people with PD [4]. Continence concerns can affect confidence with mobility and independence, such as ability to leave the house, and nocturia affects sleep. The impact of urinary symptoms is worsened by the movement disorder, with people unable to act quickly on urgency episodes contributing to incontinence, and nocturia being problematic due to PD medication being less effective at night. Urinary symptoms are strongly implicated in causing falls and contribute to the risk of hospitalisation [5], with consequent costs to the health system and wider society.

Anti-cholinergic drugs are utilised most commonly but are poorly tolerated despite evidence of their effectiveness. These cause dry-mouth and constipation (negatively impacting dopaminergic drug absorption), precipitate cognitive impairment and can precipitate falls through exaggeration of gait dysfunction [6].

Recently beta-3 agonists have been developed which do not have cognitive side effects, but their benefit can be modest and worsen supine hypertension—another non-motor feature of PD [7]. Bladder botulinum toxin injections are a relatively recent development in the field and, although not generally associated with systemic effects, are resource-intensive and require a population with mobility problems to frequently attend hospital. New approaches to treatment are therefore a high priority if they can overcome the limitations inherent in current therapies.

#### Neuromodulation for bladder symptoms

Neuromodulation has arisen as a method of controlling bladder symptoms by indirectly modulating the nerve supply that controls the bladder. Aside from implantable sacral nerve root stimulators, one method for delivering this is percutaneous tibial nerve stimulation (PTNS). This involves the application of pulsed stimulation to the tibial nerve at the level of the medial malleolus using needle electrodes. This is performed in the urology clinic requiring the patient to attend a course of treatments over several weeks. This can lead to a significant improvement in idiopathic overactive bladder symptoms [8]. The mechanism of action is unclear but may involve modulation of aberrant spinal reflexes [9]. Three studies have demonstrated efficacy of PTNS in populations with PD, with two studies proving significant improvements in urodynamic parameters [10–12]. The advantage of this approach is that it avoids the systemic and cognitive effects that are limiting factors when using pharmacological therapy.

A further evolution of PTNS has been to deliver neuromodulation via the tibial nerve transcutaneously (transcutaneous tibial nerve stimulation—TTNS). This allows non-invasive treatment in the home environment, overcoming situations where use of PTNS in PD may be limited by mobility and burden of attending hospital regularly. TTNS has been shown to be non-inferior to PTNS [13] and has been used successfully in conditions such as multiple sclerosis [14]. Preliminary studies suggest TTNS may be beneficial and well tolerated in individuals with PD [15–17], but are limited currently with regard to sample size and comparison to sham therapy. The majority of current studies exploring TTNS have utilised TENS machines (trancutaneous electrical nerve stimulation). Whilst capable of delivering effective non-invasive stimulation, these devices are complex and unwieldly. They require multiple wires to be attached and electrodes placed accurately to achieve stimulation of a specific nerve. The pulse generator units tend to be designed to provide a range of settings and often feature many buttons, dials or touch screen interfaces. This poses a significant practical difficulty for individuals who invariably have mobility or dexterity problems, as well as potential cognitive impairment.

#### Rationale for study

The importanceof bladder symptoms in individuals with Parkinson’s is evident from the scientific literature and priorities reported by patients [4]. Challenges are posed by limited tolerability and modest efficacy of current treatments. We intend to recruit participants to take part in a randomised control trial (RCT) of TTNS delivered by the Geko™ device. This is a simple self-contained device which can be easily adhered to the ankle with many advantages over the use of a TENS device which include ease of use and lack of trailing wires as a safety consideration. A pilot study has been previously performed by different authors supporting the feasibility of using the Geko™ device for treating bladder symptoms [18], assessed in a cohort with idiopathic overactive bladder and multiple sclerosis.

### Objectives {7}

#### Hypotheses

Tibial nerve stimulation using the Geko™ device will improve bladder symptoms in people with Parkinson’s disease as compared to sham (placebo) stimulation.

#### Primary objective

To assess the effect of transcutaneous tibial nerve stimulation delivered by the Geko*™* device versus sham stimulation in people with PD and bladder symptoms on the International Consultation on Incontinence Questionnaire overactive bladder (ICIQ-OAB) score.

#### Secondary objectives

To assess the effect of Geko*™* device delivered tibial nerve stimulation versus sham stimulation in people with PD with bladder symptoms on:

- Total number of incontinence episodes and extent of nocturia
- Bowel function
- Depression
- Quality of life
- Non-invasive urodynamic parameters

### Trial design {8}

**Fig. 1:**
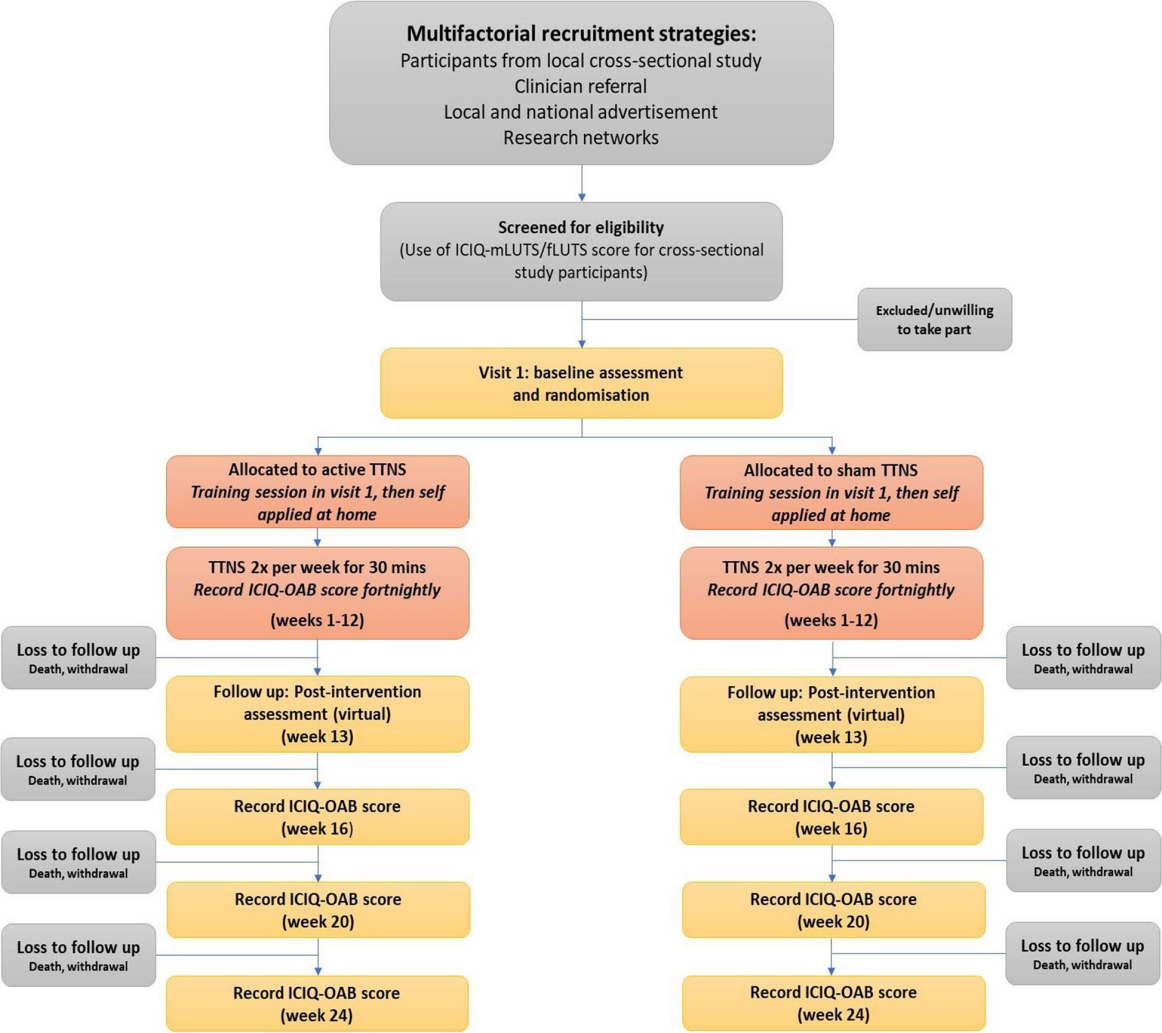
Flow chart demonstrating trial design

This study has been designed to be a phase II, randomised control trial with two arms: active stimulation at the tibial nerve and sham stimulation (allocation ratio 1:1) lasting 24 weeks. Participants will be blinded as to treatment allocation and assessment performed by participants whilst blinded. The aim of the study is to demonstrate superiority of active stimulation over sham. Figure 1 demonstrates the trial design in a flow chart format.

## Methods: participants, interventions and outcomes

### Study setting {9}

We aim to recruit 220 individuals with idiopathic Parkinson’s disease (PD) who have symptoms of urgency, nocturia or both in combination. Participants will be recruited from several sources; the PRIME-Parkinson cross-sectional study (IRAS 285,401) comprises a large sample of patients with parkinsonism across the Bath, West Wiltshire and North-East Somerset region, through advertising via research databases and via referral from appropriate clinicians throughout the UK.

In-person assessment will be carried out at the point of enrolment for baseline assessment and device training, and follow-up undertaken virtually (telephone/video call) after the intervention period for final assessment. The device will be applied and utilised by patients in their own homes between assessment visits. In person, assessments will be carried out through the Research Institute for Care of the Older Person (RICE), Bath, UK. Ongoing support whilst taking part in the trial will be provided by the team at the University of Bristol.

### Eligibility criteria {10}

#### Inclusion criteria

1. Diagnosis of idiopathic Parkinson’s disease
2. Presence of overactive bladder symptoms
3. Able to give informed consent

#### Exclusion criteria

1. Cognitive impairment to the extent of being unable to engage in assessments
2. Abnormality of both lower limbs precluding device placement on at least one ankle, including recent surgery to this region
3. Pacemaker, implanted defibrillator, sacral nerve stimulator or deep brain stimulator
4. Limited life expectancy in final palliative stages of condition
5. Alternative cause of parkinsonism including but not limited to drug-induced parkinsonism, progressive supranuclear palsy, multiple system atrophy, corticobasal degeneration and vascular parkinsonism
6. Bladder treatment medication including anti-muscarinic, beta-3 agonist, alpha-blocker, 5-alpha reductase inhibitor commenced in the 3 months prior to enrolment, or dose change within last 3 months
7. Recently diagnosed deep vein thrombosis—within the last 3 months
8. Proven urinary tract infection—within the last 1 month (new onset lower urinary tract symptoms or non-specific illness coupled with established microbial growth on a mid-stream urine specimen)
9. Current treatment for urological cancer excluding prostate cancer (unless judged suitable at the CI’s discretion)
10. Severe benign prostatic hypertrophy, based on history of predominant voiding symptoms
11. Bladder botulinum toxin injections, percutaneous tibial nerve stimulation or previous sacral nerve stimulation in the year prior to enrolment
12. Severe nocturnal polyuria (Nocturnal Polyuria index > 50%) in cases of isolated nocturia without urgency
13. Post void residual > 300 ml or residual > 100 ml with symptomatic history for incomplete bladder emptying
14. The inclusion of ‘overactive bladder symptoms’ was operationalised as patient-reported symptoms of urgency or frequency without a significant post-void residual volume demonstrated on ultrasound scan as above

### Who will take informed consent? {26a}

Written consent will be obtained for all participants by members of the research team trained in Good Clinical Practice. Verbal consent over the telephone will be obtained to collect bladder diary data prior to formal enrolment which will be used to help determine eligibility. This is intended to avoid potential participants, who are subsequently found to be unsuitable/ineligible, attending for an unnecessary in-person visit. Individuals without the capacity to make a decision to take part will not be eligible for inclusion.

### Additional consent provisions for collection and use of participant data and biological specimens {26b}

An ancillary qualitative study assessing the impact of bladder symptoms on individuals with PD will be offered to 20 participants of this study, with consent for this data to be recorded and analysed included in the STRIPE consent form.

Participants will be given the option of indicating whether their details can be retained for them to be contacted about potential future research studies that are relevant.

### Interventions

#### Explanation for the choice of comparators {6b}

**Fig. 2:**
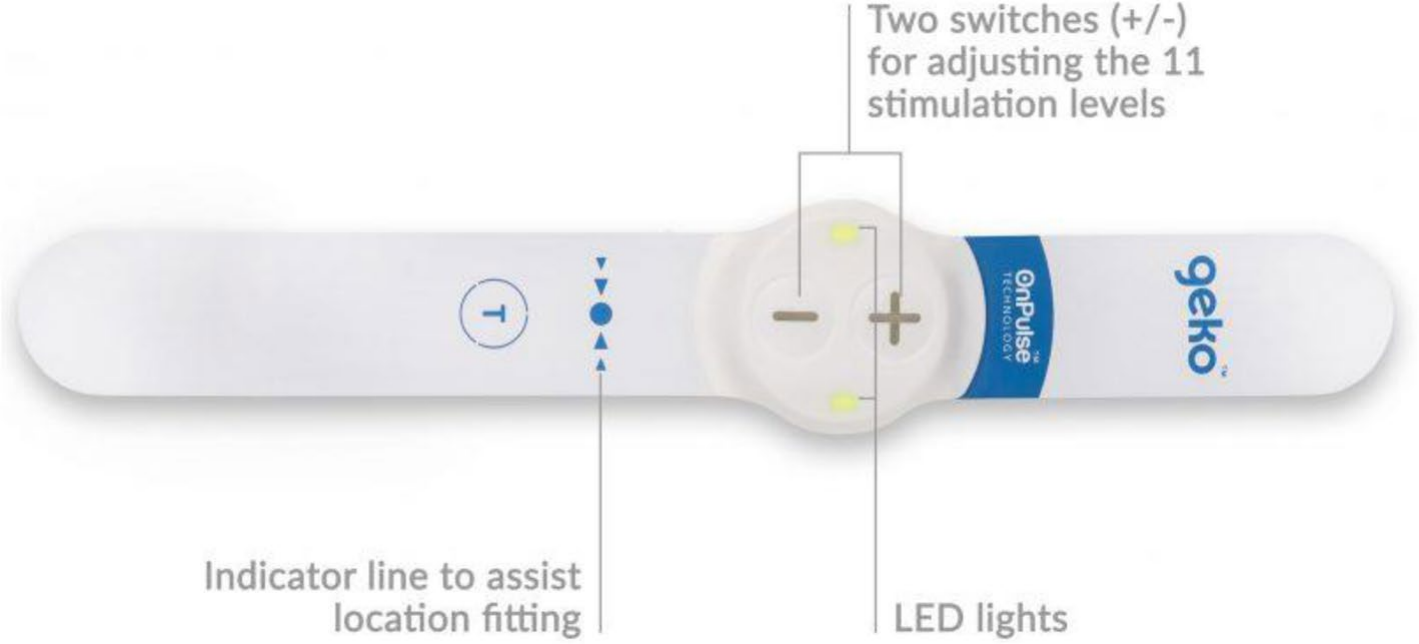
Pictorial schematic of Geko™ device, available from manufacturer’s website at https://www.gekodevices.com/geko-products/hospital-applications-device/

There is an increasing body of evidence that TTNS can provide meaningful improvement for bladder symptoms in a range of conditions, building on several decades of experience with PTNS. TTNS has the advantage of being non-invasive and simpler to use. The intervention can be used at home and has been demonstrated as non-inferior to PTNS. Where the majority of previous TTNS studies have used TENS machines, we have chosen to use the Geko™ device (Fig. 2) due to the simplicity of use. It is self-adhesive and easy to position on the ankle. It has two buttons and the only variable that can be adjusted is the pulse width (equating to energy of stimulation) using “ + ” and “- “ buttons. These also turn the device on or off if held for a prolonged period. These attributes make it ideal for use in a population with invariable dexterity issues and/or cognitive impairment. Additionally, Geko™ lacks the trailing wires leading to a stimulation unit found in a TENS device which could constitute a trip hazard in a population at risk of gait instability and falls. Geko™ has been approved for use in the UK for the prevention of DVT prophylaxis for the past decade [19], used as a neuromuscular stimulator of the common peroneal nerve to induce leg muscle contraction without mobility. A previous study in idiopathic overactive bladder and multiple sclerosis cohorts has suggested the potential use of the Geko™ device for treating bladder symptoms [18], with a dose–response relationship demonstrated (albeit inverse, assessing weekly versus daily use) suggesting biological effect and potentially a neuronal “coding” effect on bladder function.

The use of sham stimulation is extremely important in trials of medical devices and has been incorporated into this study. Previous work looking at TTNS, in particular in PD, has suggested a degree of placebo effect with sham stimulation [20], although the improvement was shown to be significantly less than the improvement seen with active stimulation. We have undertaken exploratory work to develop a sham stimulation paradigm that is believable for participants (with sensory feedback experienced) all the while minimising the risk of providing any degree of active tibial nerve stimulation.

#### Intervention description {11a}

The two arms of STRIPE comprise of an active and a sham stimulation protocol. For both arms, participants will be asked to use the Geko™ device for 30 min, twice per week, over a period of 12 weeks. Participants will be trained to use devices in-person at their baseline assessment visit. The first stimulation session will take place at this face-to-face session with training and instruction provided to equip participants to self-administer the intervention at home.

Geko™ provides neuromuscular stimulation at a fixed rate of 1 Hz with a variable pulse width of 35–560 μs using the current T3 model. A single Geko™ device will be used for four stimulation sessions (two weeks) before being replaced with a new device (to mitigate for wear of the electroconductive glue). Between uses, the Geko™ device will be stored adhered to an A4 acetate sheet.

Participants allocated to the active arm will be instructed to place the Geko™ device with the long arm immediately behind the medial malleolus, with the device running parallel along the leg and the writing upright. Stimulation will ideally be titrated to the point of motor stimulation (flexion or fanning of the toes) and participants are encouraged to proceed with the strongest level of motor stimulation that can be comfortably accommodated for 30 min. Alternatively in individuals in whom motor stimulation cannot reliably be provoked, the maximum level of tolerated sensory stimulation will be sought. An inadequate sensory response will be grounds for non-inclusion in the study.

**Fig. 3:**
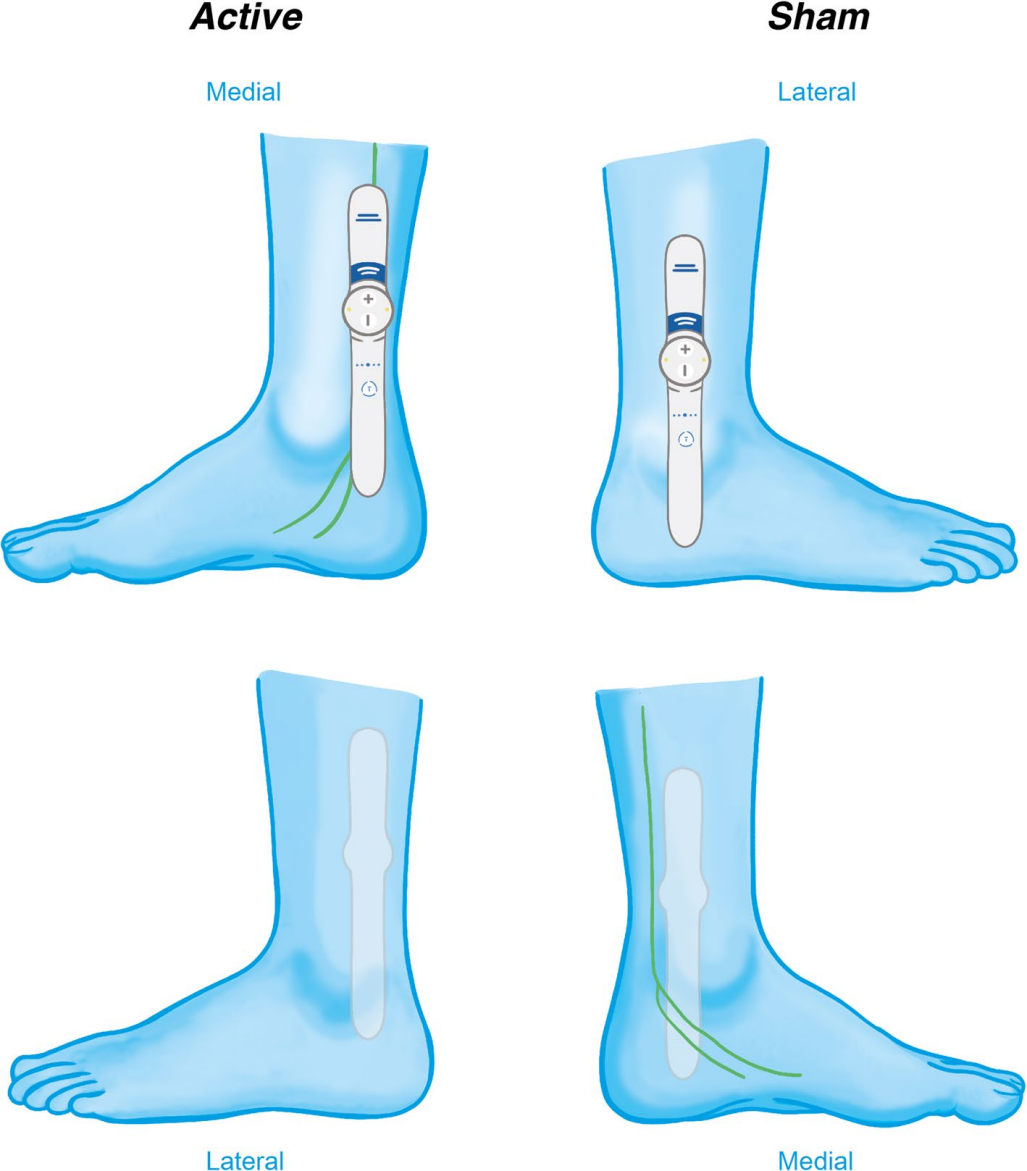
Diagram demonstrating positioning of Geko™ device for active and sham stimulation arms

Participants in the sham stimulation arm will be instructed to place the Geko™ device with the long arm on the bony prominence of the lateral malleolus and the device running parallel up the leg along the fibula. Stimulation will be left at the lowest setting, providing a slight sensation without risking depolarising any large nerve fibres. Figure 3 demonstrates visually Geko™ device positioning for each trial arm.

Participants will be encouraged to bring caregivers with them to trial visits to enable them to provide assistance where necessary to optimise concordance, e.g. in achieving correct device placement. This is particularly important because of the dexterity difficulties that occur in Parkinson’s. Therefore, caregivers will be trained along with participants in device placement.

#### Criteria for discontinuing or modifying allocated interventions {11b}

The investigators will discontinue the intervention if they deem that continuation would potentially harm or be detrimental to the participant’s health or well-being.

Reasons for discontinuation include:

- Voluntary withdrawal from the trial
- Inability to complete assessments
- Unacceptable adverse effects or intercurrent illness as determined by the investigators, in particular causing deviation from treatment schedule
- Any change to the participant’s condition justifying discontinuation of treatment

Participants who discontinue the intervention will be asked to remain in the trial and continue assessment if possible. They will be included in final data analysis.

#### Strategies to improve adherence to interventions {11c}

Participants will be assisted as much as possible to remain in the trial and with a dedicated helpline. A follow-up phone call will be undertaken after the first week to assess progress and adherence as well as answer any questions. Participants will also be given a diary booklet for them to document briefly each stimulation session, including whether this was shorter than required.

Bespoke, colour instruction sets will be provided for individuals in each arm of the trial and training sessions tailored to promote correct placement of the device for the relevant arm without compromising believability. Participants will be provided with a fridge magnet documenting the relevant placement position to remind them of optimal technique.

#### Relevant concomitant care permitted or prohibited during the trial {11d}

Pre-existing medications for bladder symptoms and other relevant treatments that may have any influence on bladder function can be continued during the trial period, as long as they are stable without dosing changes. All medications taken by a participant will be recorded in their case record file and any changes made during the period of the study confirmed. Commencing a new relevant medication within the three previous months is a criterion for exclusion but will not preclude recruitment once the 3-month timepoint is reached.

For participants who have been introduced through specialist urology services and are currently considering a specialist treatment such as bladder botulinum toxin, sacral nerve stimulation or catheterisation, recruitment into the study will be dependent on the patient agreeing that this is suspended until completion of the intervention period.

#### Provisions for post-trial care {30}

Any washout effect will be assessed using post-intervention questionnaires following the main interventional period. Usual care for participants will continue to be provided by the UK National Health Service.

### Outcomes {12}

Assessment will be obtained from a number of time points. In-person assessment will be carried out at baseline during the initial visit where written consent is formally taken and the first stimulation administered (following completion of baseline assessments). Data will be collected from questionnaires embedded in the participant stimulation diary and further post-intervention questionnaire pack. Participants will also be asked to complete a bladder diary prior to attending their initial visit, as well as in the final week of the intervention period (contained within stimulation diary). The initial bladder diary will be used as a baseline assessment, as well as a screening tool for recruitment. A second follow-up appointment will be undertaken following the intervention period, by telephone/video call and with the use of posted questionnaires and tracked delivery to collect trial materiel (stimulation diaries, follow-up questionnaires, used devices).

Basic demographic information, medication history, relevant co-morbidities and duration of diagnosis will be collected and documented in the case reference file for each participant. Each participant will also have an overall assessment of PD symptoms (Movement Disorder Society – Unified Parkinson’s Disease Rating Scale [21]; MDS-UPDRS) at baseline and lying/standing blood pressures (0- and 3-min time points) assessed at each visit. Table 1 presents a participant timeline for each assessment event.

**Table 1:**
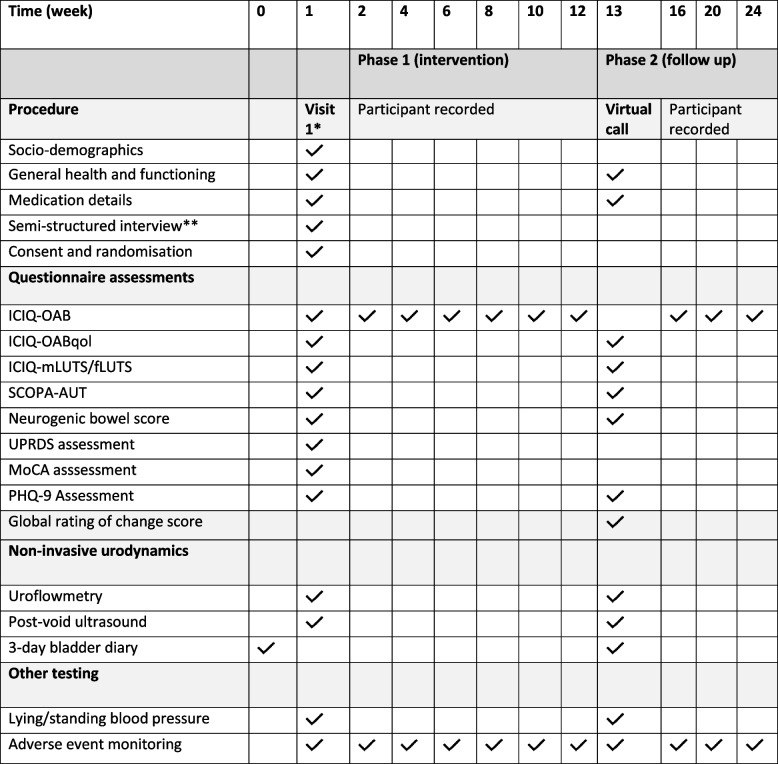
Trial assessment procedures throughout the timeline. Participants are seen in person at baseline and week 13 time points. Additional self-completed assessments occur at fortnightly intervals during the intervention period. AE monitoring is performed continually throughout the trial. The primary outcome is assessed at week 12. ^*^Assessments performed before first intervention administered. ^**^Limited to twenty participants

#### Primary outcome

The primary outcome measure will comprise ICIQ-OAB score at week 12, at the end of the interventional period.

ICIQ-OAB was chosen because it relates directly to the predominant modality of bladder symptoms in PD (overactive bladder) and the type of symptoms primarily aided by TTNS interventions. ICIQ-OAB is short to complete, comprising of only four brief questions that aid its repetition to allow monitoring over time whilst minimising participant burden. Data on minimum clinically important difference has also been established for ICIQ-OAB [22]. The ICIQ-OAB score is derived from four questions as part of the large ICIQ male/female lower urinary tract (mLUTS and fLUTS) questionnaires. These have been “recommended with caveats” for use in PD [23]. We will explore potential associations between effectiveness and factors such as disease stage, measured according to the Hoehn and Yahr score, as predefined sub-analyses.

#### Secondary outcomes

Secondary outcome measures will be taken from assessments carried out during in-person visits and bladder diaries at baseline and immediately following intervention. Importantly, this will include the difference in voiding frequency and occurrence of urgency/incontinence episodes. As part of this, participants will undergo non-invasive urodynamic measures and more detailed questionnaire assessment (see Table 2). Amongst these secondary outcomes is a Global Rating of Change Score, based on the question “Compared to before you started, how much difference have the Geko devices made to your bladder symptoms after using them for 12 weeks?”. After baseline, participants will be asked to self-complete the ICIQ-OAB questionnaire fortnightly at home whilst receiving the intervention (weeks 2, 4, 6, 8, 10 and 12) in their stimulation diary booklets. This will be returned by tracked delivery after the virtual follow-up appointment. A further ICIQ-OAB questionnaire pack will be provided to be completed at weeks 16, 20 and 24 in the post-intervention period. A further stamped addressed envelope will be included to return this to the investigators after completion.

**Table 2:** List of outcomes and assessment measures with time points

Outcome measure	Assessed by:	Assessed at:
Primary		
Overactive bladder symptoms	ICIQ-OAB part A [24]	Weeks 0–12, 16, 20, 24
Secondary		
Overall patient-perceived benefit	Global Rating of Change score (GRC)	Week 13
Bladder-related quality of life	ICIQ-OAB part B	Weeks 0–12, 16, 20, 24
	ICIQ-OABqol [25]	Week 0,13
Mood	Patient Health Questionnaire (PHQ9-) [26]	Week 0,13
General bladder symptom profile	ICIQ-mLUTS/fLUTS [24, 27] Frequency of incontinence and urgency episodes (bladder diary)	Week 0,13
	Frequency of nocturia (bladder diary)	
Autonomic symptoms	Scales for Outcomes in Parkinson’s Disease- Autonomic (SCOPA-AUT) [28]	Week 0,13
Bowel symptoms	Neurogenic bowel score [29]	Week 0,13
Urodynamic parameters	Post-void residual (ultrasound)	Week 0,13
	Nocturnal polyuria index (bladder diary)	
	Corrected Qmax (uroflowmetery)	
Tolerabillity of Geko™ device	Stimulation Diaries	Throughout intervention period

### Participant timeline {13}

Participant timeline is shown in Table 1.

### Sample size {14}

Sample size for the RCT has been calculated based on preliminary data from a paper by Seth et al. (2018). [18] This demonstrated a significant improvement with tibial nerve stimulation delivered by the Geko™ device with a standard deviation of 2.5. Using their primary outcome measure (ICIQ-OAB questionnaire part A score), which will also serve as the primary outcome for this study, we were able to estimate the required sample size of 110 individuals per trial arm (total *n*= 220). This is based on 80% power, a two-sided significance of 0.05 and ability to detect a 1-point difference in questionnaire score, which has been suggested as the minimum clinically important difference score in some preliminary work [22]. A 10% attrition rate has also been factored in.

### Recruitment {15}

Recruitment will occur from both the PRIME-Parkinson cross-sectional cohort (Bath and surrounding area) and case identification by clinicians both inside and outside of this area. Participants in PRIME-XS have been highly phenotyped including completion of ICIQ mLUTS and fLUTS questionnaire scores for males and females respectively, indicating the presence of bladder symptoms and their nature. Specific scores for urgency and nocturia symptoms will be assessed for these individuals with relevant score thresholds determined to indicate individuals with a potentially appropriate symptom profile to participate. Only PRIME-Parkinson cross-sectional participants who have actively consented to be contacted about further studies will be recruited in this manner (included in the written consent process).

Thresholds for invitation from the PRIME-Parkinson cross-sectional cohort based on ICIQ mLUTS or fLUTS score are:

- Any one of:
  - Urgency score of “most of the time” or “all of the time” (Question 7a mLUTS, 3a fLUTS)
  - Urgency score of “sometimes” with bother score of 6 or more
  - Self-reported nocturia frequency of three times a night or more and NPi of less than 50% from bladder diary (Question 14a mLUTS, 2a fLUTS)

Awareness activities for the STRIPE trial will be undertaken within the region, with all clinicians involved in caring for people with PD approached. This will include neurologists, geriatricians and PD specialist nurses, as well as urologists.

An application will be made for the trial to be advertised on the Parkinson’s UK research directory aimed at patients and awareness of the trial will be raised at research events run by the local branches of Parkinson’s UK. STRIPE will also be advertised by the social media feed of the PRIME-Parkinson portfolio of studies.

## Assignment of interventions: allocation

### Sequence generation {16a}

Eligible participants who have provided informed consent will be randomised to one of two arms (active or sham stimulation). A randomisation sequence using minimisation will be used generated using online software (Sealed Envelope Ltd, London, UK). Age category (18–64 years, > 65 years) and use of a current bladder medication will be used in the minimisation algorithm.

Pre-specified subgroup analysis will be undertaken on participants dependent on their predominant symptom type at baseline, specifically urgency versus nocturia.

### Methods in analysis to handle protocol non-adherence and any statistical methods to handle missing data {20c}

The primary analysis will relate to the primary outcome measure performed on the intention to treat (ITT) population. A secondary analysis will be performed on the per-protocol population (PP). Secondary outcome measures will also be based on the ITT population. Safety data will be analysed based on the safety population. The Statistical Analysis Plan will be written and agreed prior to data lock. Subjects who fail to complete follow-up assessments will be assessed using a series of sensitivity analyses, using techniques such as last measurement carried forward and multiple imputation methods as appropriate.

### Plans to give access to the full protocol, participant-level data and statistical code {31c}

Anonymised trial data may be available at request following completion of STRIPE and publication of results.

## Oversight and monitoring

### Composition of the coordinating centre and trial steering committee {5d}

The trial is administratively based within Population Health Sciences at Bristol Medical School, University of Bristol (UoB), UK. Activities pertaining to recruitment and data analysis will be undertaken at UoB as well as providing routine virtual support for participants. In-person activity is carried out at the RICE centre.

The trial is embedded within the wider PRIME-Parkinson programme and is overseen by the programme governance structure.

### Composition of the data monitoring committee, its role and reporting structure {21a}

Data will be monitored by an independent medical advisor.

### Adverse event reporting and harms {22}

The Geko™ device has an established safety profile with its licensed use for DVT prophylaxis with the intervention pertaining to an alternative stimulation location located rostrally in the lower limb (tibial nerve versus common peroneal nerve). Expected device-related events are discomfort during stimulation and skin irritation relating to the electroconductive glue used for device placement. We have anticipated that people with PD can experience concurrent medical problems related to Parkinson’s or comorbidities.

Adverse events (AEs) will be recorded by participants in stimulation diaries, collected at the end of the intervention period, or recorded as they are reported via the support phone line. All adverse events, regardless of seriousness, severity or presumed relationship to study device, will be recorded in the source document and the case record form (CRF), together with any measures taken, from the point of consent to the point of final data collection and end of trial (week 24). These will be collected in the participant’s stimulation diaries and also at any routine point of contact. AEs already recorded and designated as continuing will be reviewed at the subsequent assessment.

Device-related adverse events will be documented as adverse events and reported annually in accordance with reporting requirements to the MHRA and the Research Ethics Committee. Expected serious adverse events and reactions will be reported monthly to UHBW, who act on behalf of the sponsor, and to the Independent Medical Advisor.

It is feasible that a number of clinical scenarios may arise which warrant specific onward referral and action. The specific scenarios include, but are not limited to, reported haematuria, new infective symptoms suggestive of potential urinary source and painless and painful urinary retention detected at initial assessment. Suicidality may also be reported as part of the PHQ-9 questionnaire. In these scenarios, Standardised Operating Procedures will be used to assess severity and urgency and pre-agreed referral pathways will be followed. These protocols have been developed in conjunction with the urological surgery team at the Royal United Hospital in Bath, pertaining to bladder function.

### Frequency and plans for auditing trial conduct {23}

The investigators will allow monitors (on behalf of the Sponsor), persons responsible for the audit and representatives of the Ethics Committee and of the Regulatory Authorities to have direct access to source data/documents. This is reflected in the Participant Information Sheet (PIS). Study monitoring will be undertaken on behalf of the Sponsor using their monitoring standard operating procedure.

### Patient and public involvement

Specific patient and public involvement (PPI) work was carried during the set-up phase of the trial, comprising interviews with Parkinson’s patients. This included the design of the device and discussions about the intervention protocol. The approach to PPI work was modified during the pandemic but continues to ensure that the trial is designed and conducted and the results disseminated in conjunction with people affected by the condition.

### Plans for communicating important protocol amendments to relevant parties (e.g. trial participants, ethical committees) {25}

In the event of any protocol amendments, these will be submitted to the Health Research Authority providing formal authorisation to carry out the trial.

### Dissemination plans {31a}

Publication of results from this study will be undertaken in line with the University of Bristol publication guidelines. The results will be presented at conferences and peer-reviewed journals for dissemination. The results will also be made available for people with PD on the PRIME-Parkinson website and social media feed.

Participants will be provided with a plain English summary of the results at the conclusion of the trial, at the point of scientific publication.

### Implementation plan

We have not developed a comprehensive implementation plan as this is premature and will depend on whether the results appear promising or not. Assuming the data are supportive of a beneficial effect, we do not believe that this study alone will provide sufficiently robust evidence to translate directly into clinical management. We anticipate further funding being required to undertake a multi-centre trial with economic evaluation to establish a sufficient evidence base to change current guidelines and influence real-world care.

## Discussion

STRIPE aims to thoroughly assess the effectiveness of TTNS provided by the Geko™ device in a representative and realistic cohort of individuals with PD. The choice of device is based on the utility and fit with the needs of this population and should represent an elegant solution to an area in which new treatments are desperately needed.

The design of the trial is intended to determine satisfactorily whether TTNS delivered in this manner is biologically effective. The occurrence of placebo effect is an important consideration [30], particularly concerning the assessment of neuromodulation devices that act externally and do not simply disappear like a sugar pill in a drug investigation. This has proven challenging to develop a sham stimulation regime that is believable yet does not unwittingly provide active stimulation.

Most evidence for TTNS is based on historic use of PTNS, with the tibial nerve selected due to its ease of access without requiring invasive therapy. Our sham paradigm has been designed to minimise any significant depolarisation of major nerves that could mediate bladder neuromodulation effects, in particular trying to avoid influencing the sural nerve that resides laterally on the ankle. This is achieved through placement on the malleolus itself and use of the lowest setting, providing a slight sensory stimulus only and helping obfuscate the allocation status for those in the sham arm. Indeed, stimulation of other peripheral nervous targets can also have a positive effect on bladder function, highlighting the importance of ensuring stimulation outside of the active arm is minimised. Studies targeting locations such as the saphenous nerve have demonstrated urodynamic effects in animals [31]. Additionally, sacral nerve stimulation (SNS -developed contemporaneously with PTNS) has become widespread [32], providing a more durable solution to overactive bladder symptoms in comparison to the repeated trips to the urology clinic needed for PTNS. However, SNS requires invasive placement and device implantation which has limited its use in older populations with neurodegenerative disease. The development of TTNS which can be delivered non-invasively at home has now reignited neuromodulation as an option for this group.

Another persistent feature from established PTNS experience are parameters such as the frequency and interval of stimulation, which tend to involve sessional stimulation over an induction period followed by maintenance therapy which is typically monthly [33]. Frequencies of 10–20 Hz are frequently been used [8], and the protocol developed by Amarenco commonly cited [34]. The Geko™ device uses a lower frequency of 1 Hz, which deviates from the majority of PTNS and TTNS literature, however has been utilised in a repurposing study for tibial nerve stimulation by another group [18]. Here it demonstrated an inverse dose–response effect between daily and weekly use, suggesting a biological effect, although it was not tested against placebo which is the intention of STRIPE. Evidence for the effect of lower frequencies including at 1 Hz has been demonstrated in animals [31, 35], albeit at extremely high intensities. With some preliminary clinical data backed up by animal studies, our specific rationale for using the Geko™ device is its simplicity and ease of use.

Selecting a suitable population to test such an intervention is a careful balance between power to detect a small effect and gaining data on a potentially more heterogenous yet realistic study cohort. Bladder symptoms, particularly in older individuals, can be caused by a number of issues. Previously, some studies have limited themselves to women [20], presumably to limit the effect of prostatic dysfunction which is extremely common. However, by focusing on symptoms of urgency and nocturia as inclusion criteria, we anticipate the study can represent the burden of bladder dysfunction attributable to PD, with the relatively large sample size and randomisation diminishing the effect of any contributing comorbidities.

Finally, we have chosen to exclude individuals with deep brain stimulation (DBS). This is a potential limitation in that it excludes a potential pool of participants and will inevitably hold generalisation issues if TTNS is proven effective to integrate into mainstream practice. The decision was made on safety grounds, since there is little useful data on similar devices available. However, the manufacturer of Geko™ is currently running a trial of the device in a population with pacemakers (ClinicalTrials.gov Identifier: NCT04391257). The results of this could reasonably be extrapolated and tested in patients who have received DBS.

## Trial status

Ethical approval was granted by a UK Research Ethics Committee (REC) in June 2021 and full sponsorship granted in September 2021 by the University of Bristol. The current protocol is version 3.0, dated 08/02/22, last changed following an amendment approved on 28/02/22. The first participant was recruited on 25/10/21.

## Data Availability

Data may be made available on request to the Chief Investigator, Dr Emily Henderson.

## Abbreviations

CRF: Case record file
DBS: Deep brain stimulation
ITT: Intention to treat population
PD: Parkinson’s disease
PP: Per protocol population
PTNS: Percutaneous tibial nerve stimulation
REC: Research Ethics Committee
TENS: Transcutaneous electrical stimulation
TTNS: Tibial nerve stimulation
